# Gastroprotective Effects of *Spirulina platensis*, Golden Kiwifruit Flesh, and Golden Kiwifruit Peel Extracts Individually or in Combination against Indomethacin-Induced Gastric Ulcer in Rats

**DOI:** 10.3390/nu13103499

**Published:** 2021-10-03

**Authors:** Ibrahim S. Aleid, Hani A. Alfheeaid, Thamer Aljutaily, Raghad M. Alhomaid, Hend F. Alharbi, Sami A. Althwab, Hassan A. Abdel-Rahman, Metab A. AlGeffari, Hassan Barakat

**Affiliations:** 1 Department of Food Science and Human Nutrition, College of Agriculture and Veterinary Medicine, Qassim University, Buraydah 51452, Saudi Arabia; i-aleid@hotmail.com (I.S.A.); h.alfheeaid@qu.edu.sa (H.A.A.); thamer.aljutaily@qu.edu.sa (T.A.); r.alhomaid@qu.edu.sa (R.M.A.); hf.alharbi@qu.edu.sa (H.F.A.); thaoab@qu.edu.sa (S.A.A.); 2 Department of Physiology, Faculty of Veterinary Medicine, Sadat City University, Sadat City 32897, Egypt; habdelrhman59@vet.usc.edu.eg; 3 Family and Community Medicine Department, College of Medicine, Qassim University, Buraydah 51452, Saudi Arabia; m.geffari@qu.edu.sa; 4 Food Technology Department, Faculty of Agriculture, Benha University, Moshtohor 13736, Egypt

**Keywords:** *Spirulina*, Kiwifruit, Antiulcerogenic, Gastric ulcer, nutraceuticals, functional and therapeutical foods

## Abstract

This study was conducted to investigate the therapeutic effect of hydro-alcoholic extract of *Spirulina platensis* (SP), golden kiwifruit (*Actinidia chinensis*) flesh (KF), and golden kiwifruit peel (KP) individually or in combination (SFP) on indomethacin-induced gastric ulcer in rats. Negative control rats (GI) were orally administered distilled water in parallel with other treatments. The positive control rat group (GII) was administered 30 mg kg^−1^ indomethacin to induce gastric ulcers. The KF and KF extracts were used individually or together with SP in treating indomethacin-induced gastric ulcerated rat groups. Gastric ulcerated rat’s groups GIII, GIV, GV, GVI, and GVII were orally administered at 30 mg kg^−1^ rat body weight as total phenolic content (TPC) equivalent from SP, KF, KP, SPF extracts, and Lansoprazole (30 mg kg^−1^, as reference drug) daily up to 14 days, respectively. The relevant biochemical parameters, antioxidant biomarkers, and histopathological examination were examined. Remarkably, treating rats with SP, KF, KP, and SFP extracts markedly reduced gastric juice and stomach volume expansion induced by indomethacin. The SP significantly retrieved the pH of gastric juice to a regular rate compared to GI. The ulcer index (UI) was significantly attenuated by SP, KF, KP, and SFP administration. The protection index percentage (PI %) was 80.79, 54.51, 66.08, 75.74, and 74.86% in GIII, GIV, GV, GVI, and GVII, respectively. The gastric mucin content was significantly better attenuated by 95.7 in GIII compared to its content in GI. Lansoprazole increased mucin content by 80.3%, which was considerably lower than SP and SFP. SP, KF, KP, SFP, and Lansoprazole improved the reform of gastric mucosal-increased secreted mucus by 95.6, 61.3, 64.8, 103.1, and 80.2% in GIII, GIV, GV, GVI, and GVII, respectively. Interestingly, SFP efficiently increased vit. B_12_ level by 46.0% compared to other treatments. While Lansoprazole administrating did not significantly attenuate vit. B_12_ level. The SP and SFP improved iron and Hemoglobin (HB) levels depending on treatment. SP, KF, KP, and SFP significantly decreased the malondialdehyde (MDA) and increased reduced glutathione (GSH) as well as superoxide dismutase (SOD) levels in blood and stomach tissues. The most potent effect was observed with SP, and SFP was even better than Lansoprazole. Histopathologically, treating rats with SP extract showed a marked reduction of gastric damage and severity changes induced by indomethacin. KP was much better than KF in lessening gastric histopathological damages caused by indomethacin. SFP significantly alleviates gastric histopathological alterations. The lansoprazole-treated group (GVII) greatly relieved the gastric histopathological changes and recorded mild focal necrosis and desquamation of the mucosa in addition to mild oedema in the serosal layer. In conclusion, the presented results proved the antiulcer potential of SP and *A. chinensis* extracts against an indomethacin-induced gastric ulcer in rats, which may be due to their antioxidant and anti-inflammation efficiency. Thus, these data suggested that SP, KF, KP, and SFP extracts as natural and safe alternatives have a gastroprotective potential against indomethacin-induced gastric ulceration. The antioxidative and anti-inflammatory properties are probable mechanisms.

## 1. Introduction

Gastric ulceration is the most prevalent gastrointestinal disorder accounting for an estimated mortality of 15 out of every 15,000 complications yearly [1]. The most prevalent causes of gastric ulcers are *Helicobacter pylori* infection (infecting almost half of the world’s population, causes acute gastritis, chronic atrophic gastritis, gastro-esophageal reflux, ulcers of the stomach, and duodenum, esophageal cancer, gastric adenocarcinoma, MALT lymphomas, and gastric adenocarcinoma), nonsteroidal anti-inflammatory drugs (NSAIDs), and persistent drinking with gastric malignancy and chronic gastric ischemia being the less common causes. However, the clinical outcome of the infection may be influenced by a combination of bacterial factors, host factors, and environmental variables [2,3,4,5]. With regards to NSAIDs, indomethacin is a nonsteroidal anti-inflammatory drug that was introduced in 1963 to treat inflammatory diseases [6]. It is readily absorbed from the gastrointestinal tract almost entirely after oral ingestion and is metabolized by the liver and converted to active metabolites [7]. The clinical use of indomethacin is associated with potentially life-threatening deleterious effects as gastrointestinal ulceration, bleeding [8], renal toxicity [9], hepatic injury [10], intestinal damage, anemia, and the loss of protein [11]. In addition, the administration of indomethacin results in serious adverse effects on the cardiovascular system [12], initiation of lipid peroxidation, the elevation of oxidative stress [13], and infiltration of inflammatory cells [14]. 

*Spirulina platensis* (SP) is a blue-green alga found in many lakes. It contains approximately 70% easily digestible protein, where 18 out of 22 amino acids and all essential amino acids are available, making it a unique, complete protein source. Carotenoids, vitamins, minerals, and essential fatty acids are available in SP. It is an excellent source of B vitamins, particularly vitamin B_12_. This nutritious food also contains vitamin E, a highly bioavailable source of iron, 14 naturally chelated minerals, and numerous trace elements [15]. SP is claimed in folk medicine to be a potent wound-healing inducer of external and gastrointestinal wounds. Indeed, pre-clinical and clinical studies suggest it has various therapeutic effects, such as reduction in blood cholesterol; protection against some cancers [16]; enhancement of the immune system; an increase of intestinal lactobacilli, a decrease of nephrotoxicity by heavy metals and drugs; radiation protection; and reduction of hyperlipidemia and obesity [17]. 

It is evident from the scientific literature that kiwifruit has potentially beneficial actions in improving health in several domains. In that regard, ultimate biological activities toward specific diseases could be approached to recommend dietetic therapy application. There are rare or no available publications about using kiwifruit as a gastro-protective agent despite efficient application in the treatment of diabetic foot ulcers [18,19]. Kiwifruit had higher total flavonoids, total chlorophyll, carotenoids, and vitamin C [20,21,22,23]. It prevents tissue damage induced by indomethacin toxicity and protects gastric and hepatic tissues [24].

Stomach ulcers are often treated with antibiotics or medications to reduce, block, or neutralize stomach acid. The commercially available synthetic anti-ulcer-drugs are often expensive, have many side effects, and do not prevent ulcer recurrence. There has been growing interest in alternative therapies and natural products in recent years, especially those derived from plants. Foods containing the antioxidant polyphenols can protect you from ulcers and help ulcers heal, such as polyphenol-rich foods. Interestingly, there is no available information on the antiulcerogenic activity of SP and kiwifruit individually or in combination against peptic ulcers. However, even though the literature showed promising potentialities related to the use of SP and kiwifruit [18] separately, the gastroprotective potential of SP and kiwifruit both individually or in combination needs to be carefully investigated. Moreover, literature has mainly reviewed the antiulcerogenic efficiency of golden kiwifruit in Swiss albino mice [24], but the antiulcerogenic and gastroprotective potential of golden kiwifruit, their peels, and SP individually and/or in combination has not been studied so far, thus motivating this work. Therefore, the current study aims to investigate the possible antiulcerogenic and gastroprotective potential of golden kiwifruit (flesh and peel) and SP extracts against an indomethacin-induced gastric ulcer in rats model, which will be be further investiaged for potential application in functional supplements or beverages as well as in dietetic therapy for peptic and duodenum ulcers.

## 2. Materials and Methods

### 2.1. Plant-Based Materials 

Spirulina (*Spirulina platensis* Geitler) biomass powder was purchased from FRONTIER CO-OP referred to www.frontiercoop.com, accessed on 15 September 2021. Fresh kiwifruit from golden (*A. chinensis* Zesperi Sun Gold, 3279 Italy) were obtained from the local market, Riyadh city, KSA, in fresh status. 

### 2.2. Preparation of Spirulina Platensis, KF, and KP Extracts

The Spirulina was extracted using Kajimoto and Murakami [25] method with minor modifications. Dried SP was treated with 10 volumes of hydro-alcoholic solvent (ethanol:water, 1:1, *v*:*v*), then sonicated for 7.5 min. After three extractions, the extract was filtered, the filtrate was concentrated by rotary evaporator at 40 °C, and then kept undercooling for biological assessment and analysis. Kiwifruit was washed and manually peeled, cut into halves, freeze-dried (kiwi flesh and peels), and extracted with hydro-alcoholic solvent [ethanol:water, (1:1, *v*:*v*)]. Afterward, the extract was concentrated by a rotary evaporator at 40 °C. The KP extract was prepared using the same extraction solution and a similar procedure.

### 2.3. Animals

Male albino Wistar rats weighing between 180–200 g were housed at the Department of food science and human nutrition, College of Agriculture and Veterinary Medicine, Qassim University, Saudi Arabia, with hygienic conditions. Clean plastic cages constitue the animal room. The animals are allowed to acclimatize to the laboratory environment for 2 weeks under laboratory conditions of photoperiod (12-h light: 12-h dark cycle), a minimum relative humidity of 40–45%, and temperature of 23 ± 2 °C. Tap water is provided ad libitium. All rats received a commercial diet obtained from a local company. All experiments were approved by the Institutional Animal Ethics Committee (IAEC) of QU (No. 16-9-2019), KSA, which is regulated by the Purpose of the Control and the Supervision of Experiments on Animals (CPCSEA) Committee under the National Committee of BioEthics (NCBE), Implementing Regulations of the Law of Ethics of Research on Living Creatures.

### 2.4. Experimental Design

Fifty-six male albino Wistar rats were divided into seven groups (*n* = 8), as follows: GI: Negative control rats: An equal volume of distilled water was administered to the control group as a vehicle; GII: Positive control [induced gastric ulcer, receives indomethacin 30 mg kg^−1^ body weight as a single dose orally per week (Bhattacharya et al. [26]; GIII: Ulcer-induced rats receive 30 mg SP kg^−1^ bw; GIV: Ulcer-induced rats receive 30 mg KF kg^−1^ bw; GV: Ulcer-induced rats receive 30 mg KP kg^−1^ bw; GVI: Ulcer-induced rats receive 10 mg SP kg^−1^ bw, 10 mg KF kg^−1^ bw and 10 mg KPE kg^−1^ bw; and GVII: Ulcer-induced rats receive Lansoprazole (30 mg kg^−1^ bw- Ranbaxy (UK) Limited) as a references group. Briefly, rats were deprived of food but were allowed free access to water 12 h before ulcer induction. The mentioned 30 mg kg^−1^ from SP, KF, KP, and SFP is a supposed dose for oral administration similar to the dose taken from Lansoprazole daily for 14 days. The mentioned SP, KF, and KP doses are basically calculated according to the TPC of obtained extracts. 

### 2.5. Blood Collection

On the 15th day, the heparinized blood samples of different experimental rats were obtained from the cardiac puncture and centrifuged (1000 rpm at 5 °C for 10 min) immediately after collection. The plasma obtained was preserved at −18 °C until use.

### 2.6. Stomach and Collection of Gastric Juice

On the 15th day, all rat groups were killed with a high dose of thiopental sodium (50 mg/kg). The stomachs of all rats were excised after cardiac and pyloric regions were ligated. The stomach after that opened along great curvature. The gastric content was collected, centrifuged, and its volume and pH were measured. Five ml of distilled water was added, and the resultant solution was centrifuged at 3000 rpm for 10 min. The supernatant obtained after that was used for biochemical analyses. The cleaned stomachs were preserved in 0.1 M phosphate saline buffer (1:4 (*w*/*v*), pH 7.4) before macroscopic examination and homogenization [27].

### 2.7. Determination of the Ulcer and Protection Indices

Immediately after killing, the collected stomach of the rats from all the studied groups was studied to examine the ulcer index for each animal according to Nguelefack et al. [28] with advanced modification. In brief, opened stomach along great curvature was cleaned by gentle washing by 0.1 M phosphate saline buffer (pH 7.4). Afterwards, the stomach was immersed in 10 mL of 0.1% Alcian blue containing 0.16 M sucrose and 50 mM sodium acetate, pH 5.8, for 2 h. The stomach was rinsed twice with 10 mL of 0.25 M sucrose for 20 min to remove excessive Alcian dye then pinned onto a white corkboard. The mucosal lesion area (mm^2^) was measured by IMAGE-analyzer (Kodak ID program v., 3.6) using a digital Olympus camera (16 MP, *24x*) as recognized as an unstained area, see Figure S1. The Ulcer Index (UI) for each rat was determined as the mean lesion area (mm^2^), and the percentage of inhibition (PI%) was calculated using the following formula:

$$\mathrm{PI}\%=\frac{{\;\mathrm{UI}\;}_{\mathrm{Iigu}}–{\mathrm{UI}\;}_{\mathrm{Treatment}}\;}{{\mathrm{UI}}_{\;\mathrm{Iigu}}}\;\times 100$$

where UI _Iigu_ is the ulcer index of rats treated with indomethacin, and UI _Treatment_ is the ulcer index of rats treated with SP, KF, KP, SKP, or Lansoprazole.

### 2.8. Estimation of Gastric Mucus Content

The stomachs of three rats of each group were removed and opened along the great curvature. The gastric wall mucus was gently scraped with a glass slide edge and weighed according to Barka et al. [29].

### 2.9. Measurement of the Mucin Content in the Gastric Wall

Gastric mucus was quantitatively measured as described by Corne et al. [27]. The stomachs were removed and soaked in 0.1% Alcian blue solution for 2 h. The excess free dye was removed by two successive washes at 15 and 45 min in 0.25 M aqueous sucrose solution. Dye complexes with gastric wall mucous were extracted by immersion in 10 mL of 0.5 M MgCl_2_ for 2 h. The resulting blue solution was shaken with equal volumes of diethyl ether, and the optical density of the aqueous phase was measured at 605 nm by a UV–visible spectrophotometer. The quantity of mucin was expressed as grams of Alcian blue extracted per weight (g) of the stomach.

### 2.10. Gastrin Measurement

The gastrin hormone level was assayed in the collected plasma samples using an ELISA technique (Code: CSB-E12743r) according to the manual of CUSABIO TECHNOLOGY LLC (Houston, TX, USA).

### 2.11. Vitamin B_12_, Iron, and Hemoglobin Contents

Hemoglobin concentration (g dL^−1^) was colorimetrically determined in whole blood samples using Hb assay Kit (610003, MDAA Gmbh, Germany). Blood plasma was separated, then the iron and vit. B_12_ levels were determined using Cobas c311 Auto-analyzer (SN, 15A7-05, Hitachi High-Technologies Corporation, Tokyo, Japan).

### 2.12. Histological Examination 

Autopsy samples were taken from the stomach of different groups and fixed in 10% formalin saline for 24 h. Washing was done in tap water then serial dilutions of alcohol (methyl, ethyl, and absolute ethyl) were used for dehydration. Specimens were cleared in xylene and embedded in paraffin at 56 °C for 24 h. Paraffin bees wax tissue blocks were prepared for sectioning at 4 µm thickness, then stained by H&E stain for routine examination through the light microscope [30].

### 2.13. Statistical Analysis

The statistical analysis was carried out using the SPSS program (ver. 22) with multi-function utility regarding the experimental design under a significance level of 0.05 for the full results and multiple comparisons with applying LSD with Duncan [31].

## 3. Results

### 3.1. Effect of SP, KF, KP, and SFP Extracts on Gastric Physicochemical and Morphological Parameters in Indomethacin-Induced Gastric Ulcer in Rats

**Figure 1:**
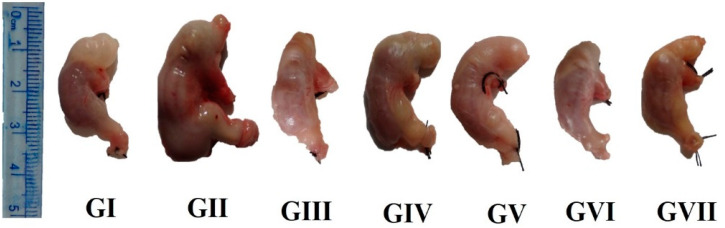
Stomach growth, appearance and volume of indomethacin-induced ulceration in rats: GI: negative control; GII: positive ulcer control; GIII: ulcer + SP; GIV: Ulcer + KF; GV: ulcer + KP; GVI: ulcer + SP, KF, KP; and GVII: ulcer+Lansoprazole (references group).

The gastric juice volume (GJV) of rats administrated only indomethacin (GII) increased significantly (*p* < 0.05) compared to normal control rats (GI) (2.13 ± 0.14 vs. 1.28 ± 0.09 mL), leading to a gastric volume increase by 66% (Table 1). In addition, the positive control (GII) group that received indomethacin was recorded with the most significant volume and congested appearance relative to other rat groups of the current study. These effects were attributed to severe bleeding due to indomethacin-induced ulceration. Therefore, this bleeding reflected on congested appearance and increased stomach volume of the GII stomach, as illustrated in Figure 1. Treating rats with SP, KF, KP, and SFP extracts markedly reduced gastric exudation and volume expansion induced by indomethacin. Notably, SP significantly decreased stomach morphometric changes induced by indomethacin (Figure 1). Giving KF, KP, and SFP extracts decreased the inflation to 11.0, 13, 5, and 25.0% in GIV, GV, and GVI when calculated based on GJV. On the contrary, the gastric juice pH decreased significantly in indomethacin-treated rats compared with the control group (2.17 ± 0.13 vs. 3.98 ± 0.05), Table 1. Interestingly, treatment with SP significantly increased the gastric juice pH to a regular rate compared to gastric juice pH in GI rats. Similarly, giving KP, KF, and SFP extracts increased the gastric juice pH of rats compared to GII rats but not usually when compared to GI. In GVII, giving Lansoprazole orally to rats caused a significant (*p* < 0.05) increase in gastric juice pH (4.68, Table 1). 

### 3.2. Effect of SP, KF, KP, and SFP Extracts on Ulcer and Protection Indexes 

As shown in Figure S1 and Table 2, no lesions and ulcers were observed in GI. Intragastric administration of indomethacin induced severe damaging and morphological changes, such as linear hemorrhages and ulceration craters in the mucosal layer (Figure S1, GII), with a significant increase of ulcer index (UI), Table 2. These changes are significantly attenuated by the administration of SP, KF, KP, and SFP for 14 days. However, SP (80.79) and SFP (75.74) were the most efficient treatments that considerably reduced areas of gastric damage (Table 2). 

The protection index (PI %) was 80.79, 54.51, 66.08, 75.74, and 74.86% for groups GIII–GVII, respectively. Remarkably, SP and SFP exhibited the most efficient therapeutic effect on rat’s gastric ulcers. Interestingly, treating rats with SP and SFP exuded a more or less similar amelioration effect as observed by using Lansoprazole (the reference drug).

### 3.3. Gastric Mucus, Mucin, and Plasma Gastrin Hormone Contents in the Stomach of Indomethacin-Induced Gastric Ulcer in Rats

Data in Table 3 illustrates the gastric juice mucin, mucus, and plasma gastrin hormone content of indomethacin-induced gastric ulcers in rats. Indomethacin-induced ulceration decreased gastric mucin content by 53.6% in GII compared to control rats (GI). Giving SP and kiwifruit extracts significantly attenuated the gastric mucin content by 95.7, 61.3, 64.7, and 103.1 in GIII, GIV, GV, and GVI, respectively, compared to regular mucin content in GI. The highest mucin-creating enhancers were SP and/or SFP, while the lowest was KF extract. Lansoprazole increased mucin content by 80.3%, which was significantly lower than SP or SFP, Table 3. As similarly shown, mucus content was dramatically decreased with inducing ulcers. The reduction was 54% in GII when compared with GI. SP, KF, KP, SFP, and Lansoprazole improved the reform of gastric mucus and increased secreted mucus by 95.6, 61.3, 64.8, 103.1, and 80.2% in GIII, GIV, GV, GVI, and GVII, respectively. Obviously, the concentration of gastrin increased in GII as a result of indomethacin administration when compared with GI. On the contrary, treating rats with SPE, KFE, KPE, SFP, and Lansoprazole significantly decreased the gastrin level in plasma. The reduction level was 64.9, 43.2, 61.8, 70.9, and 73.8% in GIII, GIV, GV, GVI, and GVII, respectively. 

### 3.4. Plasma Vitamin B_12_, Iron, and Hemoglobin Concentrations of Indomethacin-Induced Gastric Ulcer in Rats

**Figure 2:**
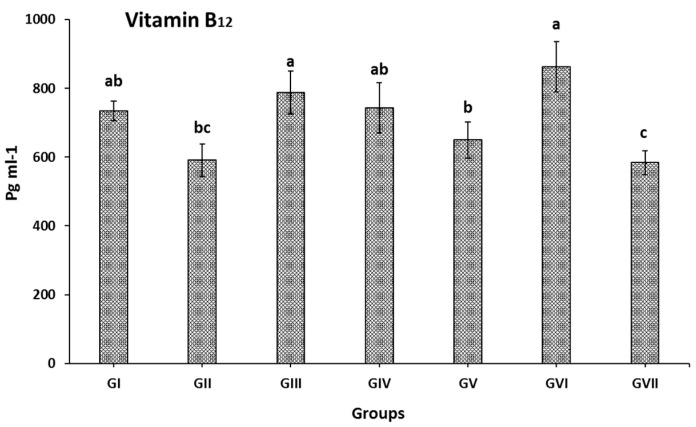
Plasma vit. B_12_ levels (pg mL^−1^) of indomethacin-induced gastric ulcer in rats: GI: negative control; GII: positive ulcer control; GIII: ulcer + SP; GIV: Ulcer+ KF; GV: ulcer + KP; GVI: ulcer + SP, KF, KP; GVII: ulcer + Lansoprazole (references group).

The effects of SP, KF, KP, and SFP extracts, and Lansoprazole on the plasma vitamin B_12_ levels of induced gastric ulcer in rats are illustrated in Figure 2. Giving indomethacin to rats significantly reduced vit. B_12_ level when compared to GI normal rats. The reduction level was 20%, from 734.8 to 591.0 pg mL^−1^ in GI and GII, respectively. In GIII, administrating SP obviously treated the induced ulcer as mentioned previously and increased vit. B_12_ level as a rich source of some vitamins, particularly vitamin B_12_. The increasing level of vit. B_12_ was 33% as it increased from 591.0 to 788.0 pg mL^−1^ in GII and GIII, respectively. Treating rats with KF and KP extracts attenuating the vit. B_12_ by 25.8 and 10.0% being KF extract was better than KP. Interestingly, administrating a combined dose of SFP increased the serum vit. B_12_ level by 46.0%, whereas it increased from 591.0 to 862.8 pg mL^−1^ in GII and GVI, respectively. On the contrary, Lansoprazole administration did not significantly attenuate vit. B_12_ level in IIGU rats as recorded in GVII rats.

Table 4 tabulated the iron (µmol L^−1^) and blood hemoglobin (g dL^−1^) levels of ulcerated rats. The serum iron and hemoglobin levels were drastically reduced when coinciding with indomethacin. The reduction rate was 22.5 and 14.4% for iron and HB levels in GII, respectively. Results showed no significant difference among GIII, GIV, GVI, and GI for iron levels. Giving Lansoprazole did not increase iron levels similarly as noticed in SP and Kiwifruit extracts. In the same context, administrating SP and fruit extracts mix with SP improved the Hb levels in ulcerated rats (Table 4). The improving percentages were 21.8, 17.0, 16.8, 22.2, and 10.9% in GIII, GIV, GV, GVI, and GVII, respectively. The lowest improvement was recorded for administrating Lansoprazole to rats.

### 3.5. Antioxidant Indices in Stomach Blood and Tissues of Indomethacin-Induced Gastric Ulcer in Rats

In the present study, administration of indomethacin resulted in a significant increase in stomach MDA and a decrease in GSH and SOD levels compared with GI rats (Table 5). Giving the indomethacin orally to the rats increased MDA significantly in GII rats compared to GI as a parallel change with ulcer induction. Interestingly, after 14 days from ulcer induction, the levels of MDA were dramatically decreased in treated groups compared with GII rats. SP, KF, KP, and SFP extracts prevented lipid peroxidation levels. Administrating SP, KF, KP, and SFP extracts significantly decreased the MDA content as showed in the treated groups. Giving SP individually or combined with KF and KP extracts was expressively better than Lansoprazole (GVII). Data in Table 5 illustrate SOD levels in the blood plasma of the studied experimental groups. Administration of indomethacin significantly decreased the levels of SOD and GSH compared with GI rats. Treatment with SP, KF, KP, and SFP extracts significantly attenuated the GSH and SOD levels. 

Data in Table 6 illustrated the MDA, SOD, and GSH levels in the stomach tissue of ulcerated rats. Administrating SP, KF, KP, and SFP extracts significantly decreased MDA and increased GSH and SOD levels in stomach tissue of treated groups, where the most preferred effect was observed with SP and SFP. Treatment with SP, KF, KP, and SFP extracts significantly attenuated the MDA, GSH, and SOD levels but were better than giving Lansoprazole. 

### 3.6. Histopathological Alterations in Rat’s Stomach

**Figure 3:**
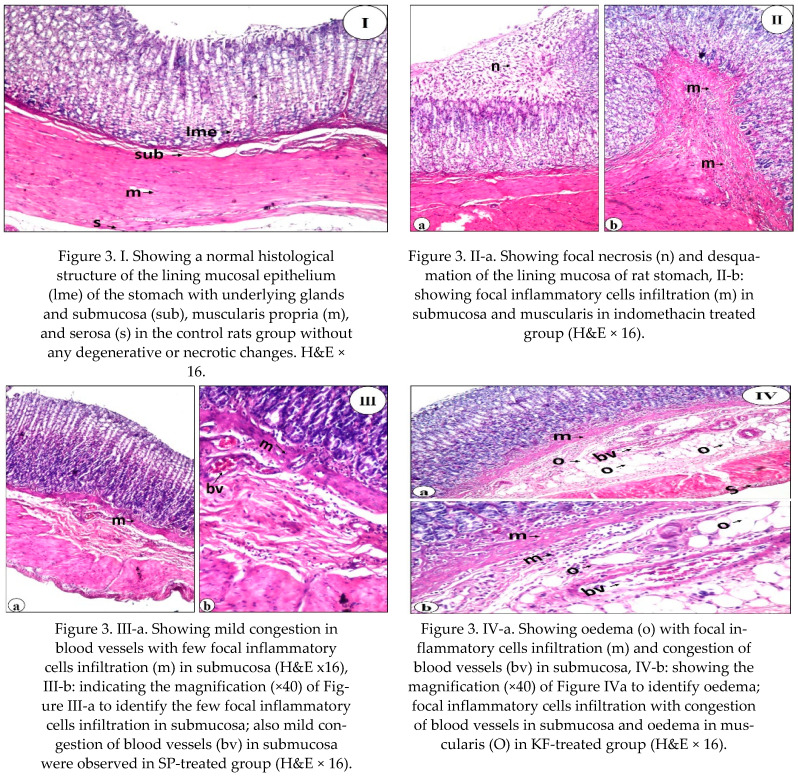

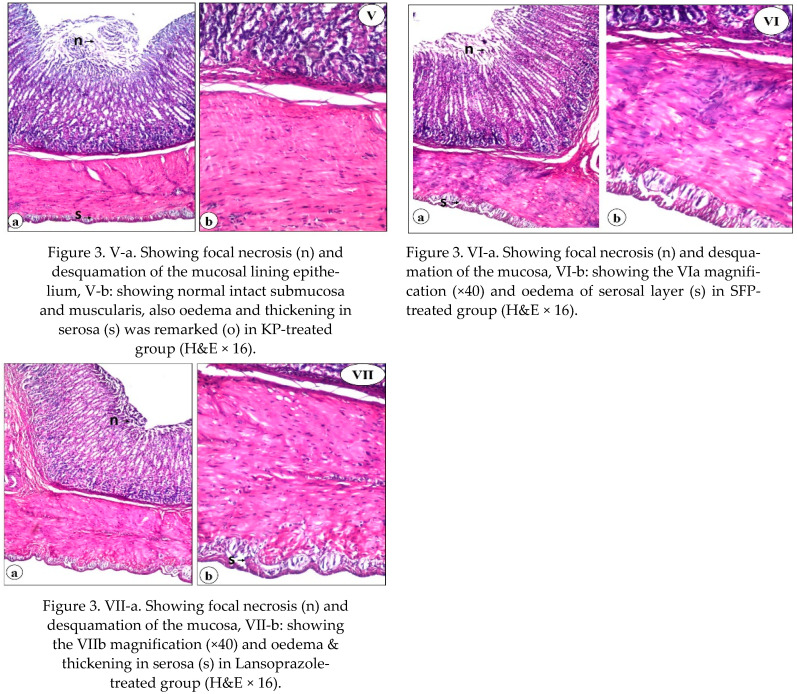
Histopathological findings of different experimental groups.

Stomach histopathological findings of control normal rats (GI) and indomethacin treated groups (II: VII) are shown in Figure 3. The severity of stomach histopathological alteration and underlying structure of different experimental groups are recorded in Table 7. There was no gastric histopathological alteration of the negative control group (GI) with the standard histological structure of the lining mucosal epithelium and the underlying glands in the lamina propria, submucosa, muscularis propria, and serosa (Figure 3I and Table 7). Rats of the positive control group (GII; indomethacin-treated) with a single dose per week, showed various degrees of pathological changes, which confirmed remarkable gastric ulceration, as proved by focal necrosis and desquamation of the mucosa (+++), focal inflammation cells infiltration in submucosa (++), and oedema of musclaris (+) and serosa (++) layers (Figure 3IIa,b and Table 7). Treating animals with 30 mg SP kg^−1^ bw showed a marked attenuation of gastric microscopic and severity changes induced by indomethacin. However, the submucosal layer showed mild (+) blood vessels congestion with mild (+) focal inflammatory cells infiltration (Figure 3IIIa,b and Table 7). The severity of histopathological alteration illustrated that SP at a given dose enhanced gastric ulcer healing relative to other experimental groups. On the contrary, oedema with focal inflammatory cells infiltration, blood vessels congestion of the submucosal layer, and oedema in the muscularis layer were detected in rats treated with KF extract (GIV) (Figure 3IVa,b). The severity of stomach histopathological alteration of GIV recorded moderate (++) focal inflammation cells infiltration and moderate (++) oedema in gastric submucosal layer, moderate (++) congestion of submucosal blood vessels, and mild (+) oedema of musclaris and serosa layers (Table 7). Administration of the KP (GV) showed moderate (++) focal necrosis and desquamation in the mucosal layer (Figure 3Va, Table 7). At the same time, the intact underlying submucosa and muscularis were improved (Figure 3Vb). Mild (+) oedema and thickening were detected in the serosa. Indeed, the KP extract was much better than the KF extract in attenuating gastric histopathological changes induced by indomethacin (Table 7). A combination of SP, KF, and KP extracts (GVI) greatly alleviated the gastric histopathological changes induced by indomethacin and recorded moderate (++) focal necrosis and desquamation of the mucosa (Figure 3VIa) and mild (+) oedema in the serosal layer (Figure 3VIb and Table 7). Also, the reference drug (Lansoprazole)-treated group (GVII) greatly relieved the gastric histopathological changes induced by indomethacin and recorded mild (+) focal necrosis and desquamation of the mucosa (Figure 3VIIa) and mild (+) oedema in the serosal layer (Figure 3VIIb and Table 7).

## 4. Discussion

Because NSAIDs are widely used worldwide due to their outstanding efficacy in managing pain, fever, and inflammation, the indomethacin model was chosen as the NSAIDs medicine. However, NSAIDs use was associated with severe adverse effects in the upper gastrointestinal tract and the small intestine, cardiovascular system, and liver; inhibited DNA synthesis; and accelerated oxidative stress in vivo [7,24,32]. Overdose, inappropriate administration, or extended usage might result in severe stomach ulcers and gastroduodenal disorders. [33]. It is believed that indomethacin inhibited prostaglandins (PGs) synthesis and blocked their therapeutic actions via inhibition of cyclooxygenase (COX) enzymes [34]. PGs biosynthesis inhibition is linked to decreased gastric mucosal blood flow; disruption of microcirculation; and decreased mucus secretion, lipid peroxidation, and neutrophil activation; all of which are implicated in the pathophysiology of gastrointestinal mucosal disorders [35]. There is a rising interest in natural antioxidants that are non-toxic, safe, and affordable, particularly those derived from plants. Natural antioxidants derived from fruits and vegetables are generally regarded as safe by most consumers [36]. Therefore, the current study compared the gastroprotective effect of SP, KF, KP, and SFP on indomethacin-toxicity in rats utilizing biochemicals and histological investigation to the commonly used Lansoprazole at equal dosages. Rats that orally received indomethacin recorded the most significant volume, congested appearance, and severe bleeding in the stomach compared to other treated rat groups after 2 weeks due to indomethacin-induced ulceration. The generation of inflammatory mediators is an essential key factor in the development of mucosal lesions. Furthermore, gastric blood flow stasis and microvascular disruption are implicated in the processes of bleeding and necrotic tissue damage [37] or as a result of stomach ischemia induced by a blockage of the stomach’s dual blood supply [2].

Neutrophil infiltration also plays an essential part in the process of injury and inflammation via aggregation and release of tissue-disrupting chemicals in numerous tissues, including stomach mucosal lesions [38]. Acute gastric mucosal lesions are caused by neutrophil infiltration into the stomach mucosal tissues [39]. Administration of SP, KF, KP, and SFP extracts markedly reduced gastric exudation and volume expansion induced by indomethacin. The best amelioration effect was observed for SP treatment (GIII). In addition, the inflation was attenuated by 83.5, 80.0, 92.9, and 62.4% when KF, KP, and SFP extracts, and Lansoprazole were given (calculated based on GJV). SP, KF, KP, and SFP showed significant inhibition of this infiltration, suggesting they possess gastroprotective properties. Similar anti-inflammatory effects were recorded with *Rhus tripartita* stem extract [29], *Ficus indica* roots [40], and *Rosmarinus officinalis* leaf extracts [41]. Giving KP, KF and SFP extracts increased the gastric juice pH of rats when compared to GII rats but not usually when compared to GI. Indeed, the significant increase of gastric pH after SP, KF, KP, and SFP extracts could be due to its inhibitory action on hydrochloric acid secretion, as similarly indicated by Barka et al. [29] and Giridharan et al. [42]. In GVII, giving Lansoprazole orally to rats recorded a significant (*p* < 0.05) increase of gastric juice pH (4.68, Table 1), which may be due to inhibition of acid secretion and protection against NSAID-induced gastric damage that depends on a reduction in mucosal oxidative injury as explained by Blandizzi et al. [43]. Inhibition of acid secretion may occur because Lansoprazole inhibits acid-secreting enzymes located in the gastric parietal cells (H+, K+-ATPase), as mentioned by Matsukawa et al. [44]. 

Regarding ulcer index and protection %, treating rats with SP and SFP exuded a more or less similar amelioration effect as observed by using Lansoprazole as treating medicine [43,44,45]. Our study demonstrated, following the literature [46], that kiwifruits have antioxidant biomolecules such as phenolics and flavonoids and exhibited antiulcer and beneficial gut health effects in vitro. In recent years, researchers indicated that various plants such as *F. indica* and *R. tripartitum* are known for their antioxidant and antiulcer therapeutic virtues [40,47]. SP exhibited gastroprotective activity against acetic acid and ethanol-induced ulcers in rats [48]. The suppressive effect of SP was similarly observed for *S. fusiformis* at 400 mg kg^−1^ as gastrointestinal ulcer treatment [42]. Recently, Guzman-Gomez et al. [49] suggested that a significant gastroprotective effect of SP was relevant to its phycobiliproteins, which repair gastric damage; its antioxidant properties by activating some enzymatic antioxidant mechanisms (SOD, CAT, and GPx); diminishing lipid peroxidation; and attenuating the inflammatory response, improving defences against the erosive lesion that characterizes the development of gastric ulcers. 

Mucosal surfaces are exposed to the external environment and pathogens. Therefore, they are protected by a secreted layer of mucus rich in mucin glycoproteins, which are the main components of mucus. Gastric mucus is an important protective factor for gastric mucosa. It consists of a viscous, elastic, adherent, and transparent gel formed by 95% water and 5% glycoproteins covering the entire gastrointestinal mucosa. It provides physical protection and hydration, excludes pathogens, and is a reservoir for antimicrobial molecules. Underlying mucus, further protection is provided by epithelial cell surface mucins, which limit microbial adherence and regulate growth and apoptosis [4]. Experimental deficiencies in mucins lead to infectious and inflammatory diseases [50]. Rat gastric mucosal damage has widely been used to investigate the gastroprotective effect of medicinal plants [51]. Ulceration induction by indomethacin decreased gastric mucin content, which significantly recovered in treated groups with SP and/or SFP better than treated with Lansoprazole. In addition to the protection function of mucus, it can act as an antioxidant and thus reduce mucosal damage mediated by oxygen-free radicals [52]. SP, KF, KP, and SFP extracts, and Lansoprazole improved the reform of gastric mucosal. The increased secreted mucus may be due to the antioxidant capacity of kiwifruit [21,22,24] and SP [42,48,49]. Gastrin is a gastrointestinal hormone that regulates gastric acid secretion, releases histamine, and regulates gastric endocrine cell proliferation [53]. Hiruma-Lima et al. [54] indicated that ulcer induction increases the gastrin level in the plasma. It is secreted by antral G cells and is the principal stimulant of gastric acid secretion, which decreases stomach pH (Table 1). On the contrary, SPE, KFE, KPE, SFP, and Lansoprazole substantially reduced plasma gastrin levels in rats [44]. Hiruma-Lima et al. [54] marked a decrease in serum gastrin level by administrating an enriched flavonoids matrix of *Alchornea castaneaefolia* hydroethanolic extract, which possesses an antiulcer mechanism. These results corroborate our present finding of an ameliorative action of SP, KF, KP, and SKP extracts on indomethacin-induced gastric ulceration, possibly due to its relatively high content of antioxidants [55]. Phytochemical analysis of SP, KF, and KP extracts indicated high phenolic acids and flavonoids content and high antioxidant capacity. Interestingly, literature reported antiulcer activity of flavonoids [54], phenolic substances [21], and bioactive compounds from herbal plants [1,40,56,57].

Treating rats with KF, KP, and SFP extracts attenuated vit. B_12_ by 25.8, 10.0, and 46.0%; SFP mix was better than both KP and KF extracts due to rich vit. C content in kiwifruit that has a positive relation to iron absorption [58] and high vitamin E. Aa highly bioavailable source of iron is SP [15]. On the other hand, administrating Lansoprazole does not significantly attenuate vit. B_12_ level in ulcerated rats as observed in GVII rats. Human studies found that oral omeprazole (treat acid reflux and ulcers) for up to 2 weeks significantly decreased vitamin B_12_ levels [59,60]. However, Kittang et al. [61] showed that an intravenous infusion of omeprazole did not change absorption of vit. B_12_ but longer treatment evidently reduced vit. B_12_ [62]. The decrease of vit B_12_ in lansoprazole-treated rats is a side effect of using such treatment. It is worth mentioning that most dietary vit. B_12_ is tightly protein bound. It is released in the stomach by gastric acid and pepsin, where it binds to salivary R proteins and intrinsic factors. This complex remains intact until it binds to specific receptors in the terminal ileum, where vit. B_12_ is absorbed [63,64]. Indomethacin significantly reduced the blood iron and hemoglobin levels. After 14 days of administrating SP and kiwifruit extracts individually or in combination, the iron level was attenuated; the result was not shown with Lansoprazole. It could be due to the efficiency of SP, KF, and SFP as an iron source (SP) and vit. C source (KF), which efficiently helps improving plasma iron level in ulcerated rats. This could explain that giving Lansoprazole did not increase iron levels similarly as noticed in SP and Kiwifruit extracts even if it treated ulcers efficiently [43,44,45]. The result is also positively correlated with obtained results in Figure 2. In the same context, administrating SP and fruit extracts mix with SP improved the Hb levels in ulcerated rats (Table 4). The lowest improving rat was observed when Lansoprazole was administered to rats, which may be related to Lansoprazole’s inefficiency in assisting the cell in absorbing iron and vitamin B_12_ as the key material for generating blood hemoglobin [59,60]. 

Indomethacin is an NSAIDS drug used as an analgesic and anti-inflammatory agent causing gastric ulcer, hepatotoxicity, and cellular damage [8,65,66]. In the present study, administration of indomethacin resulted in an increase in stomach MDA and a decrease in GSH and SOD levels significantly compared with GI rats. MDA is a secondary product of polyunsaturated fatty acids to peroxidation and is the primary marker for estimating lipid peroxidation levels [67]. Indomethacin was remarked for the initiation of lipid peroxidation [9], the elevation of oxidative stress [13], and the infiltration of inflammatory cells [14]. Interestingly, MDA levels were dramatically decreased when compared with GII rats. SP, KF, KP, and SFP extracts prevented lipid peroxidation levels, which could be attributed to the radical scavenging activity of antioxidant constituents [68]. Administrating SP individually or combined with KF and KP extracts was expressively better than Lansoprazole (GVII). However, previous studies have shown that Spirulina possesses a significant anticancer activity [69]. Giridharan et al. [42] stated that *S. fusiformis* at 400 mg/kg as gastrointestinal ulcer treatment successfully recovered peptic ulceration in rats. Spirulina contains C-phycocyanin, which is considered one of the major biliproteins. This water-soluble protein pigment is shown to have gastroprotective activity against ulcered rats with acetic acid and ethanol [48]. Recently, Guzman-Gomez et al. [49] suggested a significant gastroprotective effect of its phycobiliproteins against ethanol-induced gastric damage. This protection may be related to the antioxidant properties of phycobiliproteins by activating some enzymatic antioxidant mechanisms (SOD, CAT, and GPx), diminishing lipid peroxidation, and attenuating the inflammatory response, improving defences against the erosive lesion that characterizes the development of gastric ulcers produced by ethanol. In addition, Giridharan et al. [42] proved the hepato-renal and gastroprotective activity of *S. fusiformis* in diclofenac-treated rats. Moreover, Kepekci et al. [70] demonstrated that *S. platensis* enriched in phenolic compounds have a protective effect against hepatotoxicity induced by CCl_4_ in rats. 

The inbuilt antioxidant systems like SOD and GSH would prevent the tissues from free radical attack. Administration of indomethacin decreased the SOD and GSH levels significantly compared with GI rats, as mentioned [13,14]. GSH and SOD levels were considerably reduced after treatment with SP, KF, KP, and SFP extracts. This may be due to their content of polyphenolic compounds that may attenuate the cellular toxicity by increasing expressions of antioxidant enzymes [71], antioxidant enzymes induced by transcription factor (Nrf2) activation and other signal transduction pathways; increasing Cytochrome P450 2E1 activity (as a marker of oxidative stress);and decreasing the oxidative damage to DNA [72]. Following the known fact, the amelioration of cellular intoxication may correlate with the overall improvement of antioxidant defence mechanisms influenced by treating rats with SP, KF, KP, and SFP extracts. This increase recovered depleted GSH level and provided significant protection against GSH reduction in rats, Chu et al. [72]. Accordingly, glutathione deficiency is associated with oxidative stress and, therefore, may play a key role in the aging and pathogenesis of many diseases [73]. The possible reason is that GSH allows free radicals and ROS; consequently, its concentration decreases [69]. Therefore, supplemental ingested GSH can benefit the treatment of these diseases and increase liver GSH concentration for detoxification. Thus, it was speculated that increased GSH levels in the presence of SP, KF, KP, and SFP extracts in the face of oxidative damage enhanced the detoxification of free radical and ROS, thereby resulting in an improvement of antioxidants enzymes in rats as similarly found by various plant extracts [45,72,74,75,76]. 

As shown in the treated groups, administration of SP, KF, KP, and SFP extracts significantly decreased the MDA and increased GSH and SOD levels. The most preferred effect was observed with SP and SFP, a result that was similarly confirmed previously [69]. This suppressive effect may be due to the high content of C-phycocyanin, which is considered one of the major phycobiliproteins that have gastroprotective activity [48]. This protection may be related to the antioxidant properties of phycobiliproteins by activating some enzymatic antioxidant mechanisms (SOD, CAT, and GPx), diminishing lipid peroxidation, and attenuating the inflammatory response, improving defenses against the erosive lesion caused by gastric ulcers [70]. The inbuilt antioxidant systems like SOD and GSH would prevent the tissues from free radical attack. Administration of indomethacin decreased the SOD and GSH levels significantly compared with GI rats. Treatment with SP, KF, KP, and SFP extracts significantly attenuated the GSH and SOD levels. Their content of polyphenolic compounds may attenuate cellular toxicity by increasing the expressions of antioxidant enzymes [71]. In accordance with the known fact, the amelioration of cellular intoxication may correlate with the overall improvement of antioxidant defense mechanisms influenced by the treatments of SP, KF, KP, and SFP extracts. This increase recovered depleted the GSH level and provided significant protection against GSH reduction in rats [72]. A possible reason for this is that GSH allows free radicals and ROS; consequently, its concentration decreases [73]. Thus, it was speculated that increased GSH levels in the presence of SPE, KFE, KPE, and SFP extracts in the face of oxidative damage enhanced the detoxification of free radicals and ROS, thereby resulting in an improvement of antioxidants enzymes in rats as similarly found by various plant extracts [72,74,75,76]. Even though Lansoprazole has efficient antiulcer activity by preventing gastric mucosal from injury [45], it has no remarkable effect in enhancing antioxidants enzyme compared to SP and kiwifruit extracts. Similar results indicated that Lansoprazole has an unignorable effect on antioxidant enzymes and GSH [77]. However, the inhibition of Na^+^, K^+^ -ATPase activity can be evidence for the possible side effects of Lansoprazole when used to treat acid-dependent diseases of the stomach [78].

Using a histological examination, we evaluated the therapeutic effect of SP, KF, KP, and SFP compared to Lansoprazole. Our findings revealed that SP, KF, KP, SFP, and lansoprazole treatment improved the histological lesions. The ability of SP, KF, KP, and SFP to heal gastric ulcers in this study could be attributed to their antioxidant, anti-inflammatory, and free radical scavenging properties [24,79]. Giridharan et al. [42] explained how SP protected DFC-treated rats from oxidative stress-induced liver and kidney damage, as well as ulcer formation. The presence of phycobiliproteins in SP may explain its significant therapeutic effect. It improves defenses against the erosive lesion that characterizes the development of gastric ulcers by activating some enzymatic antioxidant mechanisms, decreasing lipid peroxidation, and attenuating the inflammatory response. Furthermore, Somchit et al. [48] reported that phyto-compounds in SP may improve wound/ulcer healing and protect the gastric mucosal layer from ulceration agents.

## 5. Conclusions

In conclusion, the presented results proved the antiulcer potential of SP and kiwifruit (*A. chinensis*) extracts against an indomethacin-induced gastric ulcer in rats due to their antioxidant and anti-inflammation efficiency. The suggested dose (30 mg kg^−1^ TPC equivalent/bw) was efficient as an antiulcer and antioxidative stress agent and is effectively comparable with Lansoprazole (30 mg kg^−1^, as a reference drug). This could be proved that using 1 mg TPC equivalent of SP, KF, KP, and SFP may be tightly correlated to 1 mg Lansoprazole. Regarding the side effects and inability of Lansoprazole to attenuate alteration in some biomarkers such as vit. B_12_, the SP and kiwifruit extracts will be promising natural alternatives for gastric ulcer treating. Further studies that regard separating the major bioactive constitutes from kiwifruits and Spirulina and studying the antiulcer and antioxidative stress activities should be deeply investigated.

## Figures and Tables

**Table 1:** Effect of SP, KF, KP, and SFP extracts on volume and pH of gastric juice in indomethacin-induced gastric ulcer in rats (mean ± SE), *n* = 6.

Physicochemical Parameters	Experimental Groups *						
	GI	GII	GIII	GIV	GV	GVI	GVII
GJ V	1.28 ^de^ ± 0.09	2.13 ^a^ ± 0.14	1.24 ^e^ ± 0.05	1.42 ^c^ ± 0.06	1.45 ^bc^ ± 0.08	1.34 ^cd^ ± 0.04	1.60 ^b^ ± 0.11
pH	3.98 ^b^ ± 0.05	2.17 ^e^ ± 0.13	4.08 ^b^ ± 0.08	3.17 ^c^ ± 0.13	2.88 ^d^ ± 0.11	3.18 ^c^ ± 0.05	4.68 ^a^ ± 0.09

GJV: gastric juice volume (mL), * GI: negative control, GII: positive ulcer control; GIII: ulcer + SP; GIV: Ulcer + KF; GV: ulcer + KP; GVI: ulcer + SFP; GVII: ulcer + Lansoprazole (reference group), ^a–e^ values with the same superscript letter in the same raw are not significantly different at *p* ≤ 0.05.

**Table 2:** Effect of SP, KF, KP, and SFP on ulcer and protection indexes of indomethacin-induced gastric ulcer in rats (mean ± SE), *n* = 6.

Items	Experimental Groups *						
	GI	GII	GIII	GIV	GV	GVI	GVII
UI [mm^2^]	0.00 ± 0.00	31.37 ^a^ ± 3.10	6.01 ^e^ ± 0.45	14.25 ^b^ ± 1.35	10.63 ^c^ ± 0.98	7.58 ^de^ ± 1.06	7.89 ^d^ ± 0.87
PI%	-	-	80.79 ^a^ ± 1.83	54.51 ^d^ ± 2.53	66.08 ^c^ ± 1.50	75.74 ^b^ ± 3.41	74.86 ^b^ ± 0.56

UI: Ulcer index, PI%: Percentage of protection index, * GI: negative control, GII: positive ulcer control; GIII: ulcer+ SP; GIV: Ulcer + KF; GV: ulcer + KP; GVI: ulcer + SFP; GVII: ulcer + Lansoprazole (references group), ^a–e^: values with the same superscript letter in the same raw are not significantly different at *p* ≤ 0.05.

**Table 3:** Effect of SP, KF, KP, and SFP extracts on gastric mucus, gastric juice mucin, and plasma gastrin of indomethacin-induced gastric ulcer in rats (mean ± SE), *n* = 3.

Items	Experimental Groups *						
	GI	GII	GIII	GIV	GV	GVI	GVII
Mucin [µg g^−1^ stomach tissue]	3.51 ^a^ ± 0.20	1.63 ^d^ ± 0.09	3.36 ^a^ ± 0.46	2.15 ^c^ ± 0.27	2.27 ^bc^ ± 0.29	3.62 ^a^ ± 0.47	2.82 ^b^ ± 0.35
Mucus [mg]	122.93 ^a^ ± 3.86	57.10 ^d^ ± 1.01	117.53^a^ ± 5.94	75.37 ^c^ ± 3.42	79.61 ^c^ ± 3.75	126.75 ^a^ ± 6.07	98.64 ^d^ ± 4.43
Gastrin [pg mL^−1^ plasma]	19.23 ^e^ ± 1.72	143.72 ^a^ ± 4.92	50.39 ^c^ ± 1.84	81.66 ^b^ ± 5.21	54.86 ^c^ ± 2.70	41.77 ^d^ ± 2.38	37.61 ^d^ ± 6.41

* GI: negative control; GII: positive ulcer control; GIII: ulcer + SP; GIV: Ulcer + KF; GV: ulcer + KP; GVI: ulcer + SFP; GVII: ulcer + Lansoprazole (references group), ^a–e^: values with the same superscript letter in the same raw are not significantly different at *p* ≤ 0.05.

**Table 4:** Effect of SP, KF, KP, and SFP extracts on blood iron and Hb concentrations of indomethacin-induced gastric ulcer in rats (mean ± SE), *n* = 6.

Physicochemical Parameters	Experimental Groups *						
	GI	GII	GIII	GIV	GV	GVI	GVII
Iron [µmol L^−1^]	28.44 ^a^ ± 3.44	22.05 ^c^ ± 1.28	30.20 ^a^ ± 1.93	29.53 ^a^ ± 2.88	23.15 ^b^ ± 3.47	31.55 ^a^ ± 2.95	24.93 ^b^ ± 1.63
Hb [g dL^−1^]	14.07 ^b^ ± 0.03	12.05 ^d^ ± 0.02	14.68 ^a^ ± 0.04	14.10 ^b^ ± 0.02	14.08 ^b^ ± 0.02	14.72 ^a^ ± 0.03	13.36 ^c^ ±0.02

* GI: negative control; GII: positive ulcer control; GIII: ulcer + SP; GIV: Ulcer + KF; GV: ulcer + KP; GVI: ulcer + SFP; GVII: ulcer + Lansoprazole (references group), ^a–e^: values with the same superscript letter in the same raw are not significantly different at *p* ≤ 0.05.

**Table 5:** Effect of SP, KF, KP, and SFP extracts on plasma antioxidants biomarkers of indomethacin-induced gastric ulcer in rats (mean ± SE), *n* = 6.

Antioxidants Biomarkers	Experimental Groups *						
	GI	GII	GIII	GIV	GV	GVI	GVII
MDA [nmol mL^−1^]	11.52 ^e^ ± 0.85	24.70 ^a^ ± 1.86	15.98 ^cd^ ± 2.33	16.04 ^cd^ ± 1.28	17.81 ^c^ ± 1.38	15.09 ^d^ ± 0.94	20.28 ^b^ ± 1.91
SOD [U L^−1^]	2.89 ^ab^ ± 0.16	1.91 ^c^ ± 0.06	3.05 ^ab^ ± 0.27	2.97 ^ab^ ± 0.26	2.81 ^ab^ ± 0.17	3.30 ^a^ ± 0.17	2.73 ^b^ ± 0.04
GSH [µmol L^−1^]	54.76 ^a^ ± 8.53	27.38 ^c^ ± 8.59	57.72 ^a^ ± 7.75	46.62 ^ab^ ± 8.00	38.85 ^b^ ± 8.44	58.09 ^a^ ± 8.62	39.59 ^ab^ ± 8.85

MDA: Malondialdehyde; SOD: Superoxide Dismutase; GSH: reduced glutathione; * GI: negative control; GII: positive ulcer control; GIII: ulcer + SP; GIV: Ulcer + KF; GV: ulcer + KP; GVI: ulcer + SFP; GVII: ulcer + Lansoprazole (references group), ^a–e^: values with the same superscript letter in the same raw are not significantly different at *p* ≤ 0.05.

**Table 6:** SPE, KFE, and KPE and their combination on stomach tissue antioxidants biomarkers of indomethacin-induced gastric ulcer in rats (mean ± SE), *n* = 3.

Antioxidants Biomarkers	Experimental Groups *						
	GI	GII	GIII	GIV	GV	GVI	GVII
MDA [nmol g^−1^ tissue]	18.00 ^bc^ ± 1.30	26.69 ^a^ ± 2.76	14.46 ^c^ ± 1.77	20.32 ^b^ ± 2.29	16.47 ^bc^ ± 1.62	13.86 ^c^ ± 3.00	20.61 ^b^ ± 1.69
SOD [U g^−1^ tissue]	14.99 ^b^ ± 0.97	4.86 ^e^ ± 0.34	13.85 ^bc^ ± 0.63	10.14 ^cd^ ± 1.26	12.38 ^bc^ ± 1.26	17.53 ^a^ ± 0.26	8.60 ^a^ ± 0.20
GSH [µmol g^−1^ tissue]	284.07 ^b^ ± 8.02	187.86 ^e^ ± 11.49	282.42 ^b^ ± 12.92	233.45 ^c^ ± 9.48	271.65 ^b^ ± 8.76	316.52 ^a^ ± 10.68	215.20 ^d^ ± 5.48

MDA: Malondialdehyde; SOD: Superoxide Dismutase; GSH: reduced glutathione; * GI: negative control; GII: positive ulcer control; GIII: ulcer + SP; GIV: Ulcer + KF; GV: ulcer + KP; GVI: ulcer + SP, KF, KP; GVII: ulcer + Lansoprazole (references group), ^a–e^: values with the same superscript letter in the same raw are not significantly different at *p* ≤ 0.05.

**Table 7:** The severity of histopathological alteration and underlying structure of stomach of different experimental groups, *n* = 6.

Histopathological Alterations	Experimental Groups *						
	GI	GII	GIII	GIV	GV	GVI	GVII
Focal necrosis and desquamation of the mucosa	-	+++	-	-	++	++	+
Focal inflammation cells infiltration in submucosa	-	++	+	++	-	-	-
Congestion in blood vessels of submucosa	-	-	+	++	-	-	-
Oedema in submucosa	-	-	-	++	-	-	-
Oedema in muscularis	-	+	-	+	-	-	-
Oedema in serosa	-	++	-	+	+	+	+

+++ = Sever, ++ = Moderate, + = Mild, - = Nil, * GI: negative control, GII: positive ulcer control; GIII: ulcer + SP; GIV: Ulcer + KF; GV: ulcer + KP; GVI: ulcer + SP, KF, KP; GVII: ulcer + Lansoprazole (references group).

## Data Availability

The data presented in this study are available on request from the corresponding author.

## Supplementary Materials

The following are available online at https://www.mdpi.com/article/10.3390/nu13103499/s1, Figure S1. Stomach ulcer morphology of indomethacin-induced ulceration in rats: GI: negative control, GII: positive ulcer control; GIII: ulcer+ SP; GIV: Ulcer+ KF; GV: ulcer+ KP; GVI: ulcer+ SFP; GVII: ulcer+ Lansoprazole (references group).

## Abbreviations

CAT: Catalase enzyme; dw: Dried weight; GPx: Glutathione peroxidase enzyme; GSH: Reduced-glutathione; KF: kiwifruit flesh; KP: kiwifruit peels; MDA: Malonaldehyde; NSAIDs: Nonsteroidal anti-inflammatory drugs; PGs: Prostaglandins; ROS: Reactive oxygen species; RSA: Radical scavenging activity; SE: Standard error; SFP: mix of SP+KF+KP in equal volumes; SOD: Superoxide dismutase; SP: *Spirulina platensis*; TPC: total phenolic content.
