# Supplementary material for: Gastroprotective Effects of Spirulina platensis, Golden Kiwifruit Flesh, and Golden Kiwifruit Peel Extracts Individually or in Combination against Indomethacin-Induced Gastric Ulcer in Rats
Source: Nutrients. 2021 Oct 3;13(10):3499. doi: 10.3390/nu13103499 (PMC8540802; doi:10.3390/nu13103499)
Supplement: Supplementary file 1 [file nutrients-13-03499-s001.zip › nutrients-1389846-supplementary.pdf]

Supplementary Figure

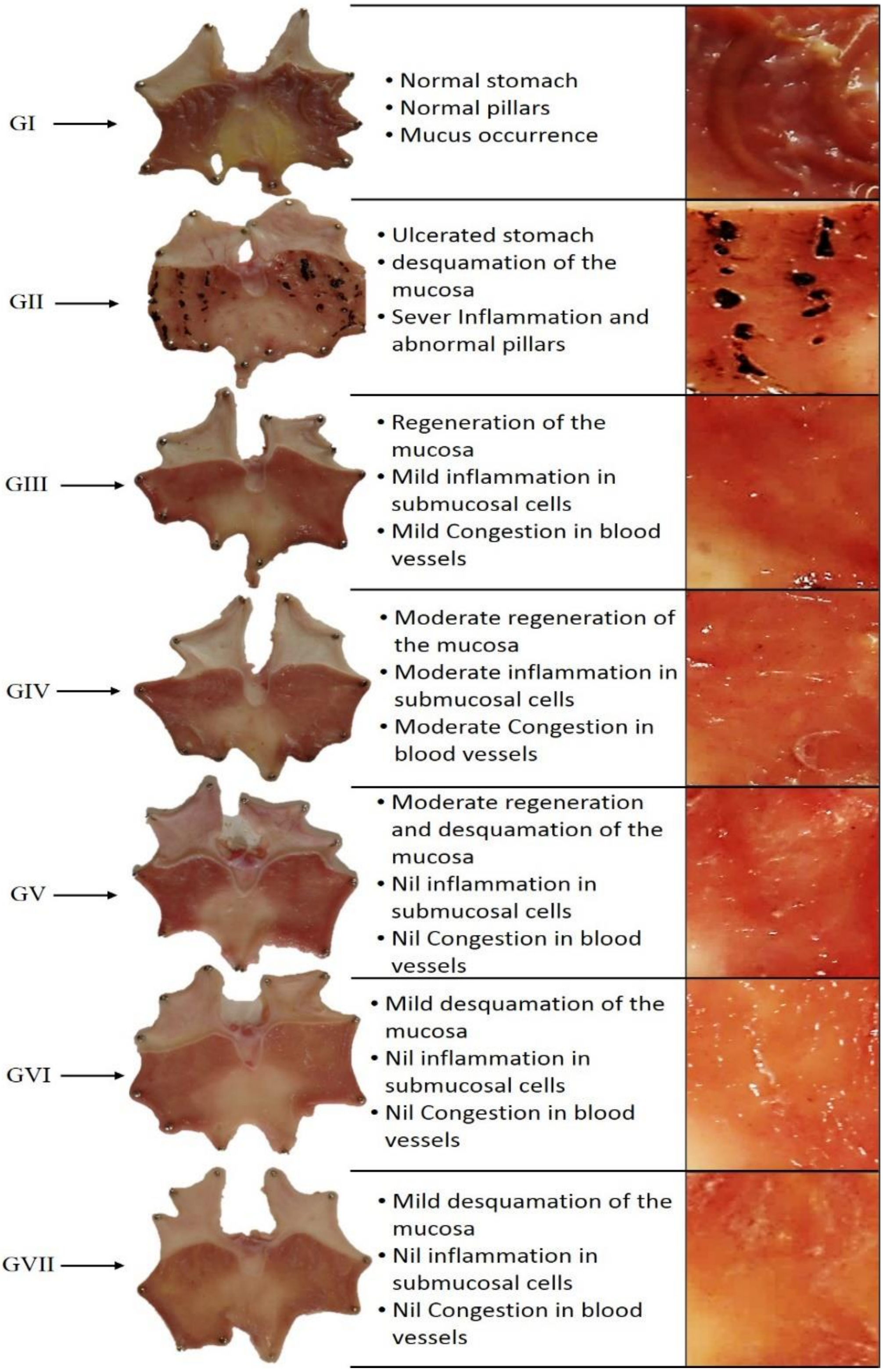

Figure S1. Stomach ulcer morphology of indomethacin-induced ulceration in rats: GI: negative control, GII: positive ulcer control; GIII: ulcer+ SP; GIV: Ulcer+ KF; GV: ulcer+ KP; GVI: ulcer+ SFP; GVII: ulcer+ Lansoprazole (references group).
